# The 2.2‐Angstrom resolution crystal structure of the carboxy‐terminal region of ataxin‐3

**DOI:** 10.1002/2211-5463.12029

**Published:** 2016-02-18

**Authors:** Vladimir A. Zhemkov, Anna A. Kulminskaya, Ilya B. Bezprozvanny, Meewhi Kim

**Affiliations:** ^1^ Laboratory of Molecular NeurodegenerationSt Petersburg State Polytechnical UniversityRussia; ^2^ Laboratory of EnzymologyNational Research Center «Kurchatov Institute»B.P. Konstantinov Petersburg Nuclear Physics InstituteGatchinaRussia; ^3^ Department of PhysiologyUniversity of Texas Southwestern Medical CenterDallasTXUSA

**Keywords:** ataxia, ataxins, Huntington's disease, polyglutamine, triplet repeat disorder

## Abstract

An expansion of polyglutamine (polyQ) sequence in ataxin‐3 protein causes spinocerebellar ataxia type 3, an inherited neurodegenerative disorder. The crystal structure of the polyQ‐containing carboxy‐terminal fragment of human ataxin‐3 was solved at 2.2‐Å resolution. The Atxn3 carboxy‐terminal fragment including 14 glutamine residues adopts both random coil and α‐helical conformations in the crystal structure. The polyQ sequence in α‐helical structure is stabilized by intrahelical hydrogen bonds mediated by glutamine side chains. The intrahelical hydrogen‐bond interactions between glutamine side chains along the axis of the polyQ α‐helix stabilize the secondary structure. Analysis of this structure furthers our understanding of the polyQ‐structural characteristics that likely underlie the pathogenesis of polyQ‐expansion disorders.

**Abbreviations**
Htt: Huntingtin
JD: Josephin domain
MBP: maltose‐binding protein
MJD/SCA3: Machado‐Joseph Disease/Spinocerebellar Ataxia Type 3
polyQ: polyglutamine
UIM: ubiquitin‐interacting motif

Machado–Joseph Disease, or Spinocerebellar Ataxia Type 3 (MJD/SCA3), is an inherited autosomal‐dominant disease of the brain that is genetically associated with CAG triplet expansion to over 55 repeats in the *ATXN3* gene 1. SCA3 shares its root cause with eight other polyglutamine (polyQ) expansion diseases 2, 3, 4. All of these disorders are transmitted in an autosomal‐dominant fashion and a major phenotype is the accumulation of polyQ‐expanded proteins or protein fragments in cells, leading to neuronal damage and brain diseases 5, 6. Ataxin‐3 (Atxn3) is a 42‐kDa evolutionarily conserved, multidomain protein that contains a polyQ tract in the C‐terminal region 7, 8. Atxn3 consists of an amino‐terminal Josephin domain (JD), an intermediate flexible linker containing two ubiquitin‐interacting motifs (UIM1 and UIM2), and a carboxy‐terminal region that includes the polyQ repeat sequence and an additional ubiquitin‐interacting motifs (UIM3) 1, 7 (Fig. 1A). Three‐dimensional structures of the JD domain have been determined by NMR spectroscopy (PDB accession codes 2AGA, 1YZB, 2DOS) 9, 10, 11 and X‐ray crystallography (3O65) 12. The UIM1–UIM2 region structure was solved by NMR (2KLZ) 13.

**Figure 1:**
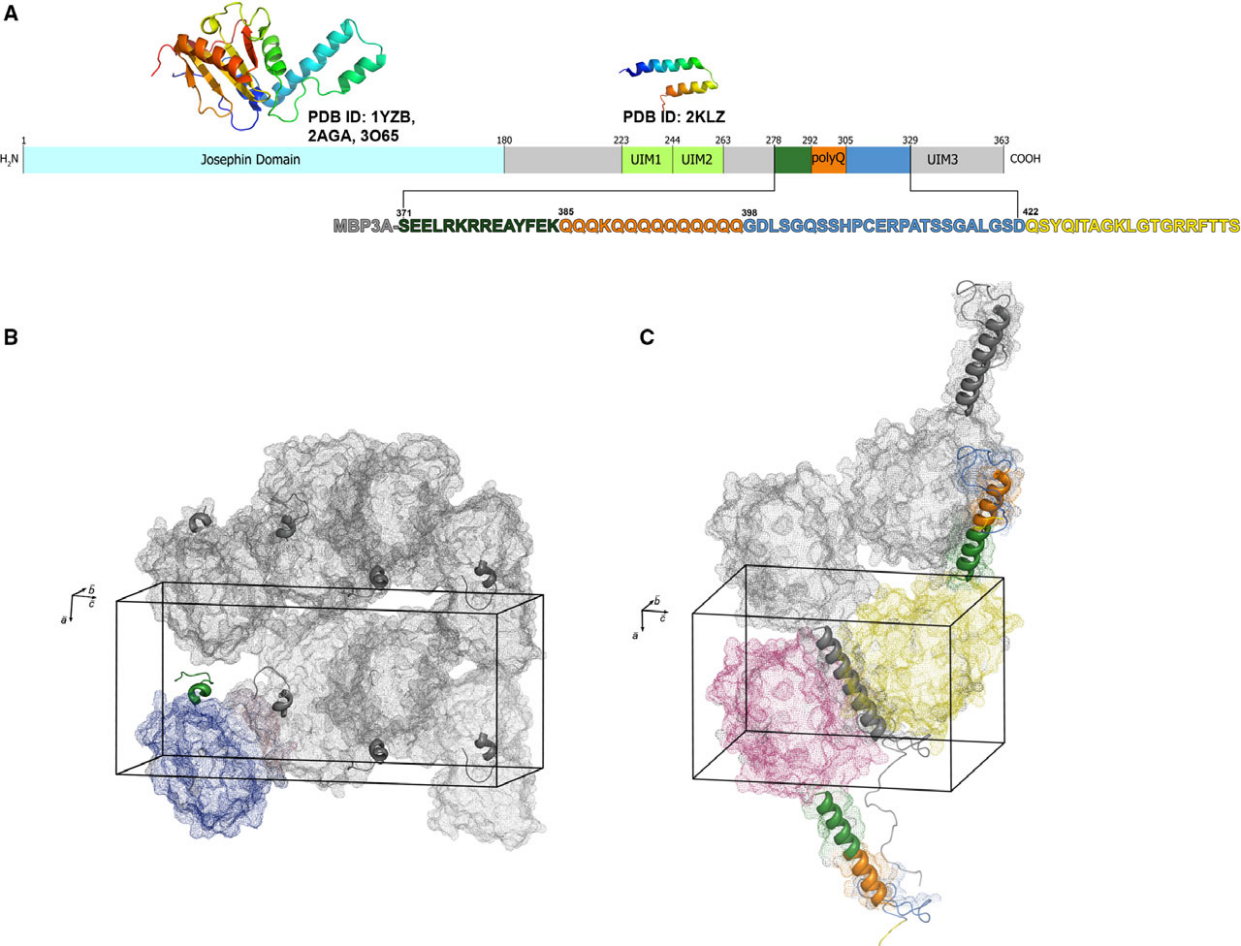
Domain structure of Atxn3 protein and crystal packing of maltose‐binding protein MBP‐Atxn3‐C in C1 and C2 crystals. (A) Domain structure of Atxn3 protein. The amino acid sequence of MBP‐Atxn3‐C expression construct is shown. MBP is shown in grey, the N‐terminal flanking sequence is in green, the polyQ stretch is in orange, the C‐terminal flanking sequence is in blue, and the C‐terminal tag added to facilitate crystallization is in yellow. (B) Packing of MBP‐Atxn3‐C in C1 crystals. (C) Packing of MBP‐Atxn3‐C in C2 crystals.

In this study we focused on structural analysis of the carboxy‐terminus of Atxn3 using X‐ray crystallography techniques. We determined the crystal structure of the Atxn3 C‐terminal region containing a polyQ repeat region with 13 glutamines interrupted by a single lysine. Both random coil and α‐helix conformations were observed in the protein crystals that diffracted at 2.2 Å resolution. Our analysis revealed an intrahelical hydrogen‐bonding pattern connecting glutamine side chains. These findings are consistent with results from our previous crystallographic studies of the Huntingtin (Htt) polyQ region 14, 15.

## Materials and methods

### Crystallization and data collection

A fragment of human Atxn3 (amino acids 278–329) containing a 14‐amino acid tract with 13 glutamines interrupted by a single lysine was amplified by polymerase chain reaction and cloned into *NotI* and *PstI* sites of the pMAL vector encoding the maltose‐binding protein (MBP) with a modified carboxy‐terminal tri‐alanine linker 16. A C‐terminal tag of 19 amino acids (QSYQITAGKLGTGRRFTTS) was added to the MBP‐Atxn3 fusion protein to facilitate crystallization 14, 15. Addition of the tag was essential in order to obtain diffracting crystals. The MBP‐Atxn3‐C fusion protein was expressed in *Escherichia coli* BL21(DE3) strain. A single colony carrying pMAL‐Atxn3‐C plasmid was inoculated into 10 mL of LB medium supplemented with ampicillin overnight at 37 °C with shaking. The culture was transferred into 1 L of LB containing ampicillin, and cells were cultivated until absorbance at 600 nm was 0.8. Protein synthesis was induced by the addition of IPTG (final concentration 0.3 mm) for 16 h at 16 °C with vigorous shaking. Then cells were harvested by centrifugation (5000 ***g***, 10 min, 4 °C) and were resuspended in 50 mL of lysis buffer (25 mm Tris‐HCl, pH 8.0, 200 mm NaCl, 1 mm DTT, 1 mm EDTA, 0.1% Triton X‐100). Cells were disrupted by sonication (three cycles for 1 min on ice, 50 : 50 pulse mode, Branson Sonifier 450). Lysate was centrifuged (70 000 ***g***, 1 h, 4 °C) and the supernatant was applied onto 3 mL of amylose resin (NEB) pre‐equilibrated with equilibration buffer (25 mm Tris‐HCl, pH 8.0, 200 mm NaCl, 1 mm DTT, 1 mm EDTA). After washing, protein was eluted with elution buffer (25 mm Tris‐HCl, pH 8.0, 200 mm NaCl, 1 mm DTT, 1 mm EDTA, 15 mm maltose). MBP‐Atxn3‐C was concentrated and applied onto the gel‐filtration column Superdex75 16/60 (GE Health Care, Piscataway, NJ, USA) pre‐equilibrated with gel‐filtration buffer (25 mm Tris‐HCl, pH 8.0, 100 mm NaCl, 1 mm DTT, 0.5 mm EDTA, 0.02% NaN_3_, 1 mm maltose) at 1 mL·min^−1^ flow rate. Fractions containing MBP‐Atxn3‐C fusion were pooled and concentrated to approximately 10 mg·mL^−1^ using Amicon centrifugal filters (Millipore Ltd, Cork, Ireland, 30000 Da MWCO). The resulting protein was at least 95% pure as shown by Coomassie staining of a SDS/PAGE. Purified MBP‐Atxn3‐C was crystallized using the hanging drop vapor diffusion technique without removing either MBP or the C‐terminal tag. Single crystals were obtained in the following crystallization conditions: 25% polyethylene glycol monomethyl ether (5K), 1.0 m sodium acetate, 0.1 m imidazole, pH 8.0, 0.1 m zinc acetate (crystal C1) and 24% polyethylene glycol monomethyl ether (5K), 0.9 m sodium acetate, 0.06 m imidazole pH 8.0, 0.1 m zinc acetate (crystal C2). No additional cryoprotection steps were undertaken due to the high polyethylene glycol concentration in the crystallization drop. Crystals were flash‐cooled in liquid nitrogen, and the complete diffraction datasets for crystals C1 and C2, which had different shapes, were collected at the 19ID beamline of Advanced Photon Source Synchrotron. These raw diffraction data were indexed and scaled using hkl‐2000 software 17.

### Structure determination

Phasing was performed by the molecular replacement method in phaser software 18 using 40 MBP PDB models from Protein Data Bank. The best solutions for datasets produced by Phaser with MBP structure (PDB ID 1ANF) were used for Atxn3‐C structure model. Structure determination was performed separately for each crystal. Electron density in the region corresponding to MBP was well defined, whereas the initial map of the Atxn3‐C region was of poor quality. The complete maps were obtained for MBP region, and partial maps were obtained for the Atxn3‐C region. The map derived from the C1 crystal dataset allowed only limited determination of Atxn3‐C structure due to intrinsic disorder of entire Atxn3‐C region. Maps derived from C2 crystals were of better quality and enabled more extensive model building. In the process of the model building and refinement *R(work)*,* R(free)* improved and the electron density of the Atxn3‐C region became clearer. The Cα backbone was built first, and side chains in the Atxn3‐C region were assigned in the later stages of refinement. Crystallographic parameters for C1 and C2 crystals and final refinement statistics are listed in Table 1. Ramachadran statistics for C2 crystal are as follows: favored 98.12%, outliers 0.13% (Table 1). The models of MBP‐Atxn3‐C in the two crystals and for molecules within the unit cell were built separately but simultaneously using coot software 19. Refinement was performed using refmac5 program 20 and validation was performed using molprobity software 21. Molecular interfaces were analyzed with pisa software 22. An OMIT map of the model and real‐space correlation coefficients were used to validate the determined Atxn3‐C structure (phenix, 23). Hydrogen bonds were identified using hbond server and secondary structure was assigned with DSSP algorithm 24, 25.

**Table 1:** Data collection and refinement statistics

	C2 (PDB: 4WTH)	C1 (PDB: 4YS9)
Data collection		
Diffraction source	APS synchrotron, 19ID beamline	
Wavelength (Å)	0.9795	
Temperature (K)	100	
Detector	Quantum 315r	
Space group	*P1*	*P14*_*1*_ *1*
Cell dimensions		
*a*,* b*,* c* (Å)	49.01, 59.77, 77.79	59.68, 59.68, 135.17
α, β, γ (°)	90.00, 89.99, 87.50	90.00, 90.00, 90.00
Resolution range (Å)	38.74–2.25 (2.33–2.25)^a^	37.65–2.00 (2.05–2.00)^a^
No. of unique reflections	39 115	31 861
*R*_sym_	0.047 (0.917)	0.053 (0.566)
Ι/σΙ	15.7 (1.2)	23.2 (1.2)
Completeness (%)	94.0 (76.1)	85.2 (58.0)
Redundancy	1.9 (1.8)	1.9 (1.3)
Refinement		
Resolution (Å)	38.74–2.25 (2.33–2.25)	37.65–2.00 (2.05–2.00)
No. of reflections, working set	37158 (2105)	30210 (2205)
No. of reflections, test set	1980 (113)	1607 (124)
Final *R*_work_	0.202 (0.541)	0.207 (0.221)
Final *R*_free_	0.249 (0.591)	0.225 (0.327)
No. of non‐H atoms		
Protein	6295	2975
Ligand/ion	7 (Zn), 46 (MAL)	4 (Zn), 23 (MAL)
Water	155	90
Average B‐factors (Å^2^)		
Protein	44.7	45.4
Ligand/ion	61.7 (Zn), 33.4 (MAL)	59.8 (Zn), 30.5 (MAL)
Water	40.0	39.4
R.m.s. deviations		
Bond lengths (Å)	0.007	0.006
Bond angles (°)	1.131	1.043
Ramachandran plot		
Most favored (%)	98.12	96.60
Allowed (%)	1.75	2.88
Outlier (%)	0.13	0.52

a Values in parentheses correspond to high‐resolution shell.

## Results and discussion

### Crystal structures of Atxn3‐C

To crystallize the carboxy‐terminal fragment of Atxn3, we adopted the crystallization approach used successfully in our previous crystallographic studies of the polyQ region of Htt (Htt‐Ex1) 14, 15. The sequence encoding human Atxn3 from S278 through D329 containing a 14‐amino acid tract with 13 glutamines interrupted by a single lysine (K388) was cloned into MBP expression vector, pMAL (see Materials and methods section for details). This poly14Q tract sequence is the one most frequently observed in normal individuals 26, 27, 28. The importance of the polyQ flanking sequences on polyQ structure, function, and toxicity have been addressed in many studies 8, 29, 30, 31, 32, 33, 34, thus we decided to express, purify, and crystallize the polyQ region of Atxn3 in its native context. The fragment we crystallized starts with S278; this residue is slightly upstream of the predicted boundary for the coiled‐coil domain (L281‐K291) 35. The protein construct ends at position D329, which is located just in front of the third UIM (G331‐T348). The structure of the UIM3 region was determined previously 13. As in our studies of Htt‐Ex1 14, 15, we added a 19‐amino acid carboxy‐terminal tag (indicated by yellow in Fig. 1A) to facilitate crystallization. Although the C‐terminal tag was not resolved in the electron density map, only constructs having this C‐tag yielded crystals. The MBP‐Atxn3‐C protein was expressed in bacteria, purified, and crystallized using the procedures reported previously 14, 15 (see Methods for details). Crystals of MBP‐Atxn3‐C were obtained in two different shapes: a prism shape (C1, tetragonal) and a pyramid shape (C2, triclinic). Crystals in both shapes diffracted with up to 2 Å resolution but the unit cell parameters were different (Table 1). The C1 crystal contained one molecule of MBP‐Atxn3‐C in the asymmetric unit (Fig. 1B), whereas the C2 crystal contained two of the molecules (Fig. 1C).

**Figure 2:**
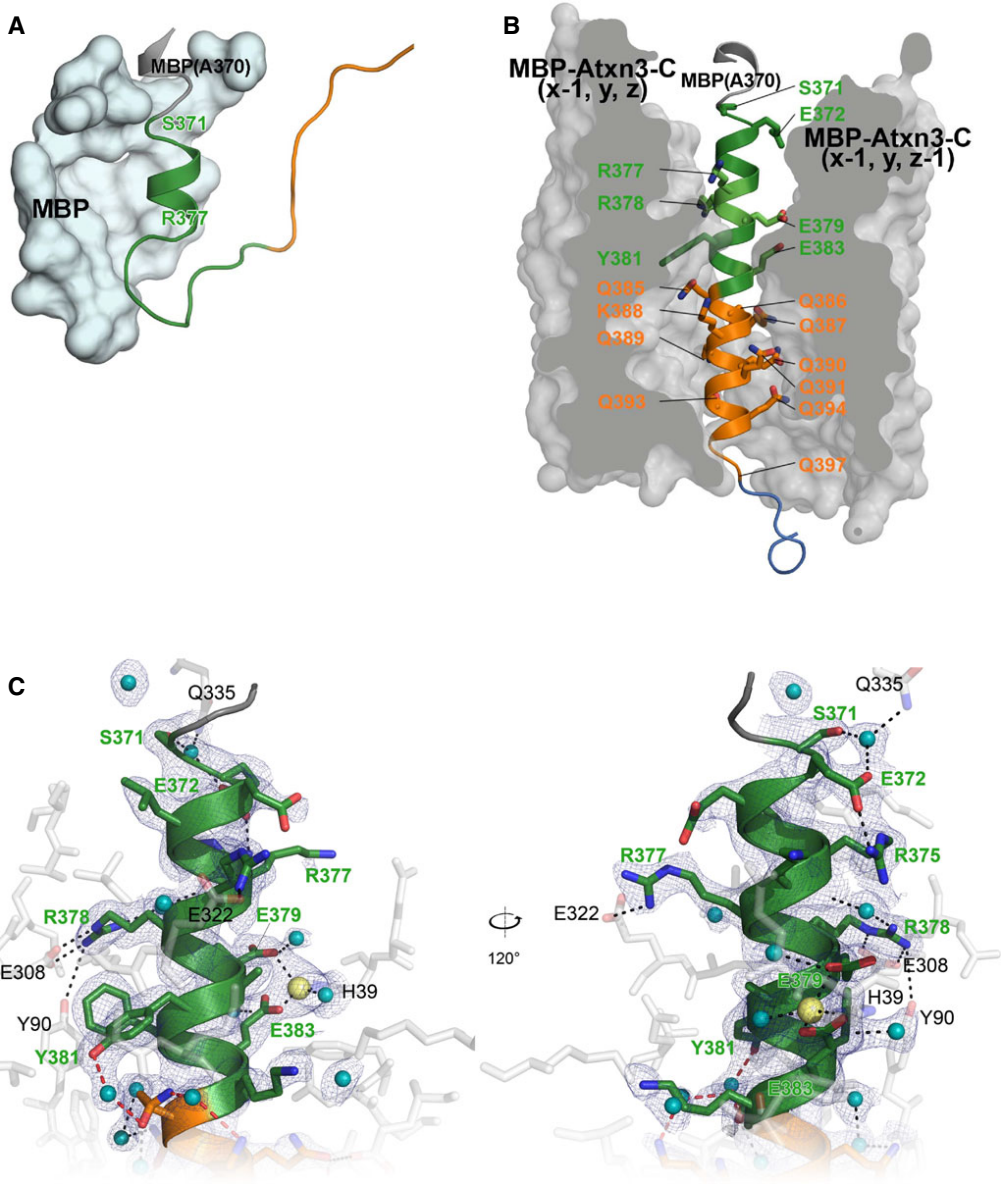
Atxn3 structure in C1 and C2 crystals and N‐terminal flanking domain of Atxn3 from C2 crystal. (A) Atxn3‐C structure in the C1 crystal. The short helical N‐terminal sequence is in green. Maltose‐binding protein (MBP) is shown as the grey surface. (B) Atxn3‐C structure in the C2 crystal. The N‐terminal flanking domain is shown in green, the polyQ tract in orange, and the C‐terminal domain in blue. The two MBP surfaces are shown in grey. (C) Atxn3 N‐terminal flanking region is shown in green. MBP residues are shown in grey. Polar contacts are indicated by black dashed lines and residues involved in interactions are labeled. The corresponding regions of electron density maps (1σ) are shown in blue mesh. Water molecules are in cyan. Left and right panels show views of the molecule differing by 120° rotation.

Structures of MBP‐Atxn3‐C in C1 and C2 crystals were solved by molecular replacement with the MBP structure as a search model (see Materials and methods section for details). The structure of the Atxn3‐C portion in the C1 crystal is illustrated on Fig. 2A. The region of Atxn3‐C was exposed to solvent. Seven residues (S371‐R377) were resolved, and these form two turns of α‐helix (Fig. 2A). A model for remaining amino acids could not be built due to insufficient electron density (Fig. 2A). The similar lack of electron density was observed for some Htt polyQ region crystals 14, 15.

In contrast to C1 crystals, the Atxn3‐C regions in the C2 crystals are partially shielded from solvent by symmetry‐related molecules of MBP (x‐1, y, z) and (x‐1, y, z‐1) (Figs 1C and 2B). The amino acids from S371 to Q397 in both of the molecules of the Atxn3‐C in the asymmetric units of the C2 crystals exist in α‐helical conformation (Fig. 2B). The crystal structure of the Atxn3 α‐helical region was solved at 2.2 Å resolution in the C2 crystals (refinement statistics in Table 1). The Atxn3‐C α‐helix forms several hydrophilic interactions with the fused MBP protein. These interactions are mediated by side chains of the N‐terminal amino acids (Fig 2B,C; residues in green) and by the polyQ residues (Fig. 2B; orange). R377 and R378 form polar contacts with MBP residues E322, E308, and Y90; E379 and E383 are involved in coordination of a Zn^2+^ ion, which bridges to H39 of MBP; and S371 and E372 form hydrogen bonds with Q335 of MBP via water molecules (Fig. 2C).

### Structure of polyQ helix

**Figure 3:**
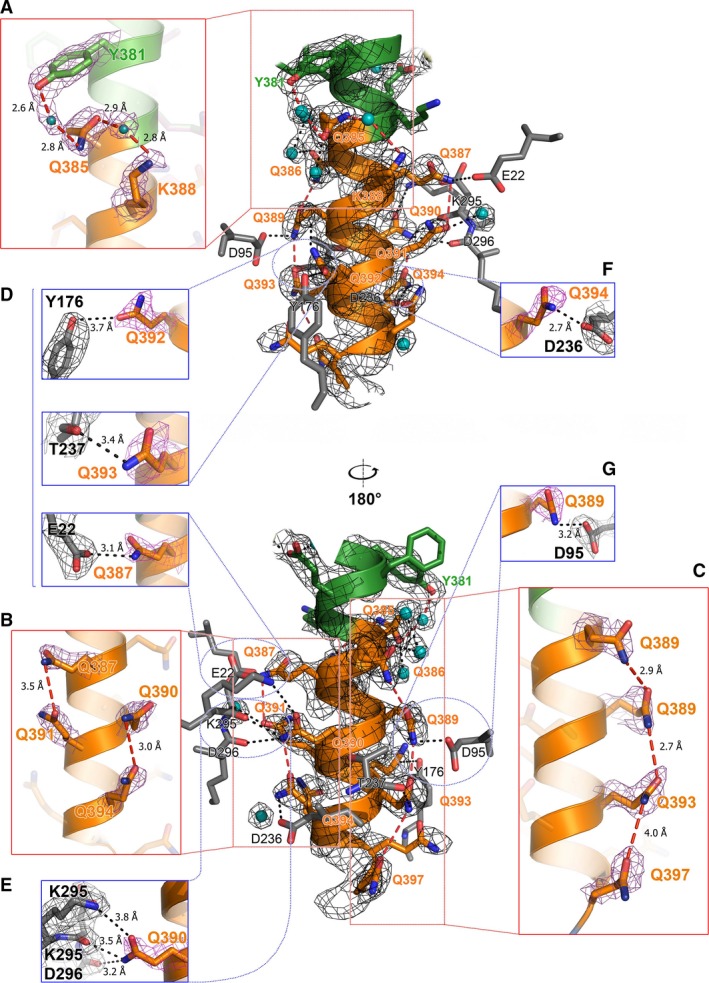
Polyglutamine structure in C2 crystal. The polyQ region of maltose‐binding protein MBP‐Atxn3‐C is shown as an orange ribbon‐stick model. N‐terminal residues are in green and residues from MBP are shown in grey. Water molecules are in cyan. Polar contacts are indicated with black dashed lines. Intramolecular hydrogen bonds between glutamine side chains are indicated with red dashed lines. 2F_o_ – F_c_ electron density maps (at 1σ) are shown by blue mesh. Upper panel shows the structure rotated by 180° relative to the lower panel. Inserts boxed in red show intrahelical interactions with OMIT map in mesh (at 0.9σ): (A) Y381–Q385–K388; (B) Q387–Q391 and Q390–Q394; (C) Q386–Q389–Q393–Q397. Inserts boxed in blue show interactions of glutamines (orange stick) with MBP residues (grey stick) with OMIT map in mesh (at 0.9σ): (D) Q392–Y176, Q393–T237, Q387–E22; (E) Q390–K295/K295/D296; (F) Q394–D236; and (G) Q389–D95.

The glutamine side chains of the polyQ α‐helix in the C2 Atxn3‐C structure form hydrogen bonds with various partners. In addition, several residues within the polyQ helix form polar contacts with MBP molecule. These interactions involve Q387, Q389, Q390, Q392, Q393, and Q394 residues and are almost exclusively with charged residues of MBP (Fig. 3): carboxy groups of aspartic or glutamic acid residues of MBP (Q387(NE2)···E22(OE2), Q389(NE2)···D95(OD2), Q394(NE2)···D236(OD2)) and amines of lysine (Q390(OE2)···K295(NZ)). Interestingly, a proteomic study of polyQ‐interacting proteins revealed enrichment in charged amino acids E, D, and K 36. In addition, the glutamine side chain of residue Q392 pairs with the less polar hydroxyl group of residue Y176 of MBP (Q392(O)···Y176(OH)). Amine groups of glutamine also form hydrogen bonds with the carboxyl groups of the backbone in MBP (Q390(NE2)···D296(O)/K295(O); Q393(NE2)···T237(O)). Several glutamine residues (Q391, Q392, Q385, Q386) form bonds with hydroxyl groups of the water molecules (Fig. 3).

**Figure 4:**
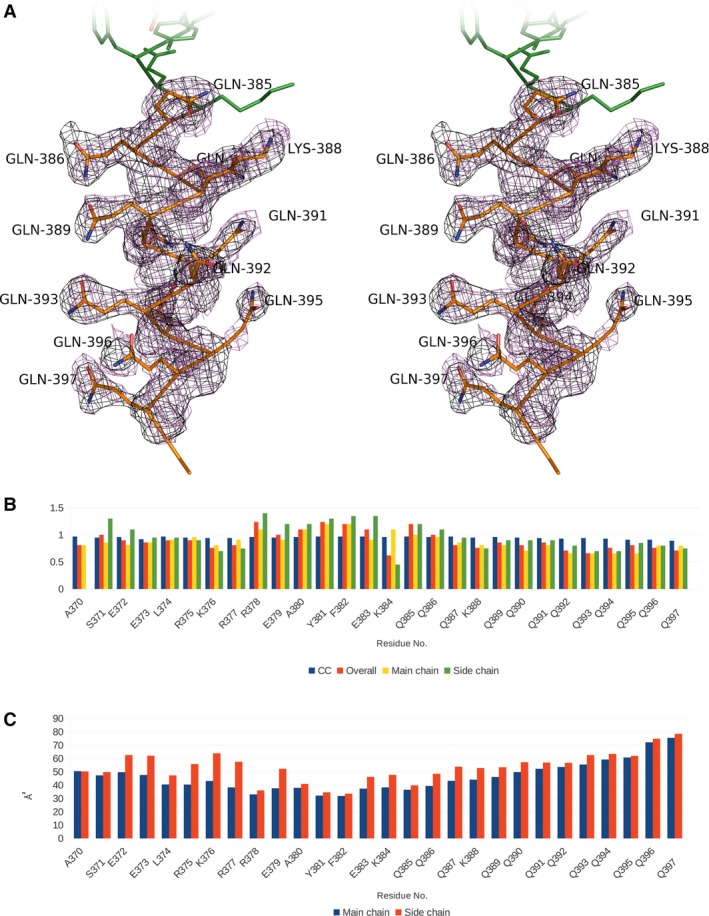
Validation of polyglutamine structure in C2 crystal. (A) Stereo image of polyQ tract structure and electron density map. The 2F_o_ – F_c_ map is shown in magenta. 2F_o_ – F_c_ OMIT map (side chains of glutamine residues omitted from calculations) is shown in white. (B) Per‐residue real‐space correlation plot for Atxn3‐C region. Blue bars indicate real‐space correlation coefficients. Electron density for each of the residue (red bars) were calculated separately from those of main‐chain atoms (yellow bars) and amino acid radicals (green bars). These numbers were normalized to the mean electron density per residue. All numbers were calculated in Phenix.map_to_model software 23. (C) Per‐residue B‐factor plot for Atxn3‐C region. B‐factors of main‐chain atoms are represented by blue bars and B‐factors of side‐chain atoms are represented by red bars.

The polyQ α‐helix of Atxn3‐C is also stabilized by interactions between glutamine resides. Eight of the 13 glutamines are involved in the formation of direct intrahelical hydrogen bonds between residue i and residue i + 4 (in four cases) or between i and i + 3 (one case) (Table 2; Fig. 3). In addition to these direct glutamine–glutamine side chain interactions, Q385 interacts nonglutamine residues at i–4 (Y381) and i + 3 (K388) via a water‐mediated bond (Table 2, Fig. 3). The polar moment of most glutamine side chains aligns in parallel with the α‐helical axis and hydrogen bonds are formed parallel to the helical axis (Figs 3 and Fig. 4A). The hydrogens of the amines in glutamine side chains are the donors and that the carboxyl oxygens are acceptors; however, the exact orientation of the amide and carbonyl groups in the glutamines could not be determined and the orientation of Q–Q pair interaction can be flipped by rotating side chain by 180 degrees without affecting the bonding pattern. Only Q392 is not involved in intrahelical interaction; it interacts with Y176 of MBP.

**Table 2:** Hydrogen bonding interaction within polyQ helix of Atxn3‐C in C2 crystal

Hydrogen bonds within polyQ region		
Residue 1 – Atxn3	Residue 2 – Atxn3	Interaction; Distance (Å)
Y381 (OH)	Q385 (OE1)	Y381…HOH; 2.6 HOH…Q385; 2.8
Q385 (NE2)	K388 (NZ)	Q385…HOH; 2.9 HOH…K388; 2.8
Q387 (NE2)	Q391 (OE1)	3.5
Q386 (NE2)	Q389 (OE1)	2.9
Q389 (NE2)	Q393 (OE1)	2.7
Q393 (NE2)	Q397 (OE1)	4.0
Q390 (NE2)	Q394 (OE1)	3.0

In the two molecules composing the asymmetric units of the C2 crystal, the glutamine side chains adopt slightly different conformations. The major difference is that in one the Q393···Q397 bond is present and in the other it is not. We calculated electron density maps with side chains omitted for both units and calculated a real‐space correlation coefficient between the map and the model (Fig. 4B) and B‐factors for main‐chain and side‐chain atoms (Fig. 4C). The modeled side chains show good quality of fit except at Q390 and Q394, which are resolved at lower resolution than the rest of the polyQ region. To summarize, the α‐helical structure of the Atxn3 polyQ region is stabilized by two kinds of hydrogen bonds, those mediated by main‐chain atoms and those between by glutamine side chains. The polyQ α‐helix is likely to be more thermodynamically stable due to the glutamine side chain‐mediated hydrogen bonds. This observation agrees with the conclusions from the host‐guest modeling study by Roy and Dannenberg 37.

### Comparison with other polyQ structures

In our previous structural studies of Htt, we observed both α‐helix and a random coil conformation of the polyQ region (PDB entries 3IOR, 3IOT, 3IOU, 3IOV, 3IOW, 3IO4, 3IO6 and 4FE8, 4FEB, 4FEC, 4FED) 14, 15. The Htt‐17Q and Htt‐36Q structures were determined at 3.5 Å and 3.0 Å resolutions, respectively 14, 15. The side chains of the glutamine residues in the Htt‐Ex1 structure were unresolved likely due to multiple conformations in the crystal. The conformational heterogeneity observed for the Htt‐Ex1 structure in a single crystal was manifested in two different crystals for Atxn3‐polyQ. Our hypothesis that the polyQ regions of Htt and Atxn3 exist in equilibrium between α‐helical and random coil conformations is supported by structural studies of polyQ repeats in which structural flexibility was observed 30, 35, 38, 39, 40, 41, 42, 43, 44.

In our analysis of Atxn3‐C, one cannot ignore the influence of MBP and crystallization effect. The stabilizing effect of MBP on the α‐helix may be due to both solvation effects and molecular interactions. In the well‐defined region from Q385 to Q394 in the C2 crystal MBP has a caging effect. MBP limits solvent access and favors intramolecular interactions between glutamines. Existing structural and biochemical data support the idea that polyQ itself is capable of forming an α‐helix. For example, a similar helical structure was observed in the polyQ region of Htt‐Ex 1 without stabilizing interactions with other regions of the crystallized protein 14, 15. Circular dichroism studies, molecular modeling, and simulations also predict the presence of α‐helix in polyQ repeats 38, 41. Glutamine has a high propensity for incorporation into helical motifs (e.g., see 45, 46, 47, 48, 49, 50). Glutamine can form side chain‐side chain bonds 48, 49. A recent modeling study provides a direct evidence for Q–Q side chain stabilization along an α‐helix 37. Both α‐helix and random coils formed by polyQ regions were also observed by circular dichroism and mutagenesis studies by Fiumara *et al*., Pelassa *et al*., and Kokona and colleagues supported the observation 35, 44, 50. A ‘conformational stabilization’ was also observed in the high‐resolution crystal structure of a 10Q peptide complexed with MW1 antibody 51. In this structure, the 10Q peptide adopts an extended conformation and is tightly bound to the MW1 antibody (PDB entries 2OTU, 2OTW). Structures of the apo‐forms of other anti‐polyQ antibodies also suggest that they recognize a similar extended polyQ conformation (4DCQ, 3S96, 4JJ5, 4ISV) 52. Interactions between polyQ and MW1 involve both side‐ and main‐chain atoms of the polyQ peptide. Nine of the glutamine side chains in 10Q are involved in interactions with the MW1 residues and backbones of each of the 10 glutamines contact MW1, indicating a very strong interaction between the peptide and the antibody 51. We reason that the MW1 antibody likely selects one of the numerous potential conformations adopted by polyQ peptides. In contrast, the Atxn3‐polyQ region forms only a few interactions with MBP cage (Figs 2B and 3), suggesting that the stabilization effect is not as stable in this crystal.

Intramolecular stabilization of a polyQ α‐helix was previously proposed 53. In this model, side chain‐mediated stabilization involves i and i + 4 glutamine residues. This spacing of side‐chain interactions was also discussed by Monoi and co‐workers 54, 55. A crystal structure of glutamine‐rich domain of HDAC4 revealed the importance of Q–Q interactions both on helical stability and helix bundle formation 56. A more recent modeling study by Roy and Dannenberg is in the excellent agreement with our experimental findings 37. In this host–guest modeling approach, regularly spaced (i, i + 4) glutamine residues were inserted into an otherwise alanine α‐helix. These glutamine substitutions stabilize the α‐helical structure, but the effect of water was not carefully examined. Our high‐resolution structure (Fig. 3) suggests that glutamines can form hydrogen bonds within the α‐helix with residues at either i + 4 or i + 3. The presence of both types of interaction leads to an interruption in the hydrogen‐bonding network. This is contradictory to the predicted end‐to‐end hydrogen‐bond array along the α‐helix 53.

The mechanism of polyQ aggregation is not apparent from our findings because the current research was focused on the polyQ tract of nonpathogenic length. It has been proposed that the polyQ α‐helix is involved in helix‐helix interactions that lead to pathogenic fibril‐aggregation in neurons 35, 57. Interestingly, in the article of Fiumara *et al*. the region of the Atxn3 sequence from L281 to K291 was predicted to be a part of an α‐helix in agreement with our crystallographic data. In comparison to solution study on the synthetic polyQ peptides and native/pathogenic Htt exon I, the studies found the polyQ in solution exists in an equilibrium between random coil and alpha‐helix (estimated helix content 40–75%) 35, 44, 50 meanwhile our study found both random coil and helical structures in each of two different crystals. Pelassa and colleagues proposed a model where random‐coil polyQ adopts an α‐helical structure upon a binding to its natural partners 44. However, further studies are needed to confirm the correlation between the structural studies and biological functions.

In the MBP‐Atxn3‐C crystal structure, glutamine residues form multiple coordinated hydrogen bonds along the polyQ helix and limited interactions with MBP. Interestingly, Perutz initially proposed a model of polyQ known as a ‘polar zipper’ model 58 where Q–Q bonds stabilize a beta‐sheet structure. The Atxn3‐C structure and the polar zipper model both show that their secondary conformations could be stabilized by Q–Q interaction. This effect would likely be less pronounced in solution due to solvation by water molecules. However, when the solvent effect is diminished, such as when the local concentration of glutamine is high due to expansion of polyQ length or accumulation of proteasome‐resistant polyQ fragments, the Q–Q stabilizing effect could lead to formation of aggregates. The exclusion of solvent could lead to self‐association of glutamines, either inter‐ or intramolecularly, further lowering the efficiency of proteasomal degradation of such oligomers and accelerating macro‐aggregation of polyQ proteins.

## Conclusion

Here, we observed that the polyQ regions of Atxn3 adopt random coil and α‐helical conformations in two separate crystal structures. The presence of both random coil and α‐helical conformations in the polyQ region is consistent with the result from our previous crystallographic studies of Htt‐Ex1 14. The analysis of the polyQ α‐helix in the MBP‐Atxn3‐C structure revealed intrahelical hydrogen bonds between glutamines along the helix axis. Such intramolecular Q–Q interactions were previously predicted to occur in both α‐helix and β‐sheet conformations of polyQ peptides. The information obtained from analysis of the polyQ domain of Atxn3 suggests that intrahelical hydrogen bonds between glutamines lead to the aggregation that is presumed to underlie the pathogenic mechanism of polyQ‐expansion proteins.

## Author contributions

MWK designed the experiments. MWK performed the crystallization and data collection experiments. VZ and MWK processed and analyzed the data. VZ, MWK, AK, IB wrote the final manuscript. All authors read and approved the final manuscript.
